# Supplementary material for: Nanoparticles suppress fluid instabilities in the thermal drawing of ultralong nanowires
Source: Nat Commun. 2020 Nov 23;11:5932. doi: 10.1038/s41467-020-19796-5 (PMC7683681; doi:10.1038/s41467-020-19796-5)
Supplement: Supplementary file 1 — Supplementary Information [file 41467_2020_19796_MOESM1_ESM.pdf]

Supplementary Information

Nanoparticles suppress fluid instabilities in the thermal drawing of ultralong nanowires

Injoo Hwang†, Zeyi Guan†, Chezheng Cao, Wenliang Tang, Chi On Chui, and Xiaochun Li*.
Correspondence to: xcli@seas.ucla.edu

Supplementary Figure 1. Function plots of $(1-x^2)\Phi(x)$ at different viscosity ratios. Under the ideal static condition, increased viscosity ratio (μ_{core}/μ_{clad}) results in decreased $G = \max\left[(1-x^2)\Phi\left(x,\frac{\mu_{core}}{\mu_{clad}}\right)\right]$, indicated by red circles in each plot.

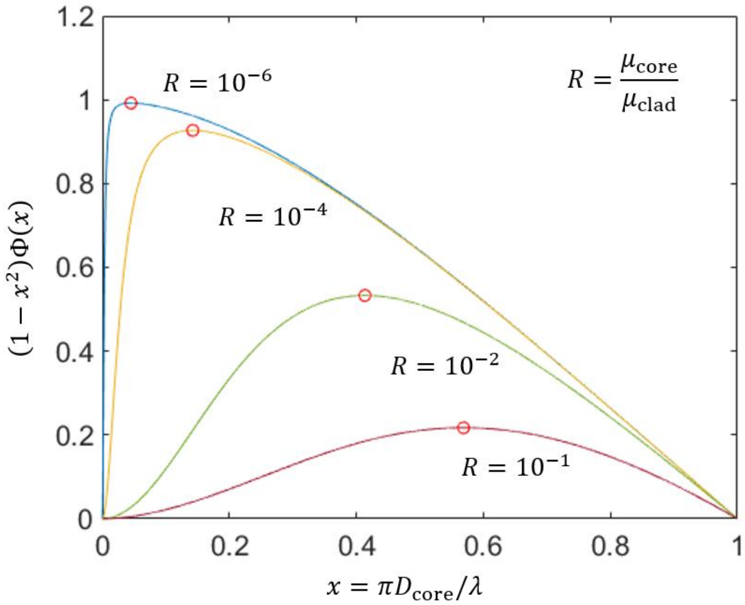

Supplementary Figure 2. Contact angle measurement. Contact angles were measured by optical microscopy for (a) Zn/borosilicate and (b) Zn-10WC/ borosilicate at a temperature of 800°C.

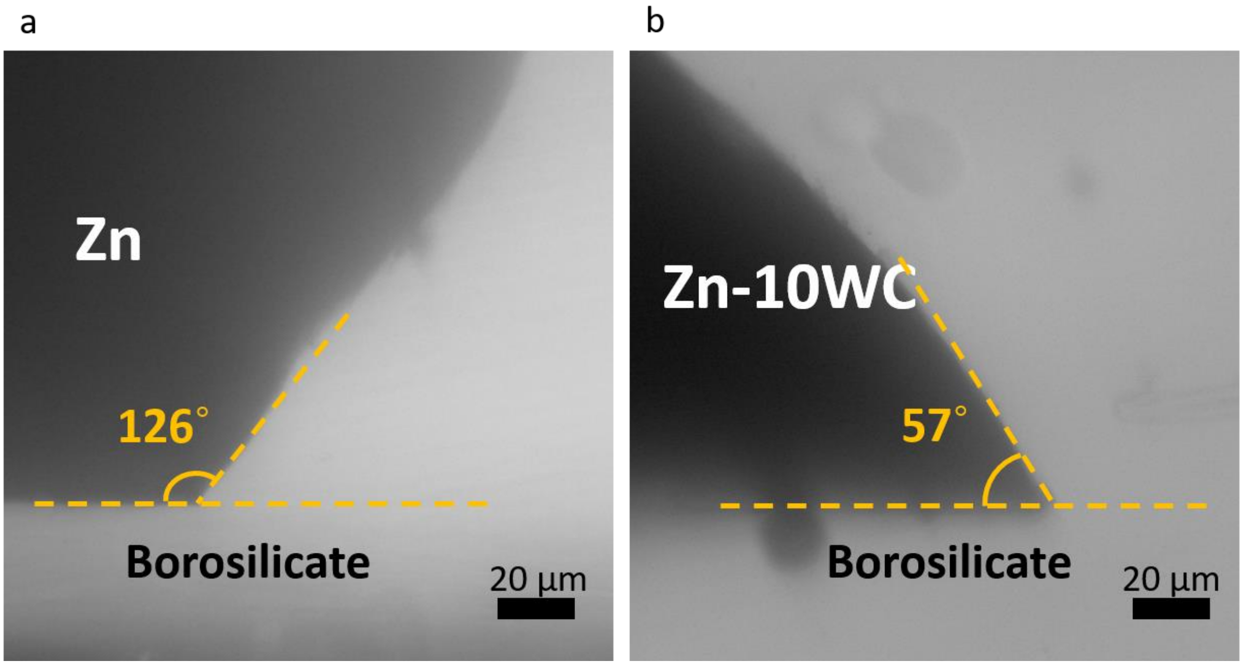

Supplementary Figure 3. Schematic of the thermal fiber drawing tower. Metal wire embedded in borosilicate was fed into the high temperature furnace by the feeding motor with a speed of $\boldsymbol{v}_{\mathbf{f}}$, and was pulled out of the furnace by the pulling roller with a speed of the $\boldsymbol{v}_{\mathbf{p}}$.

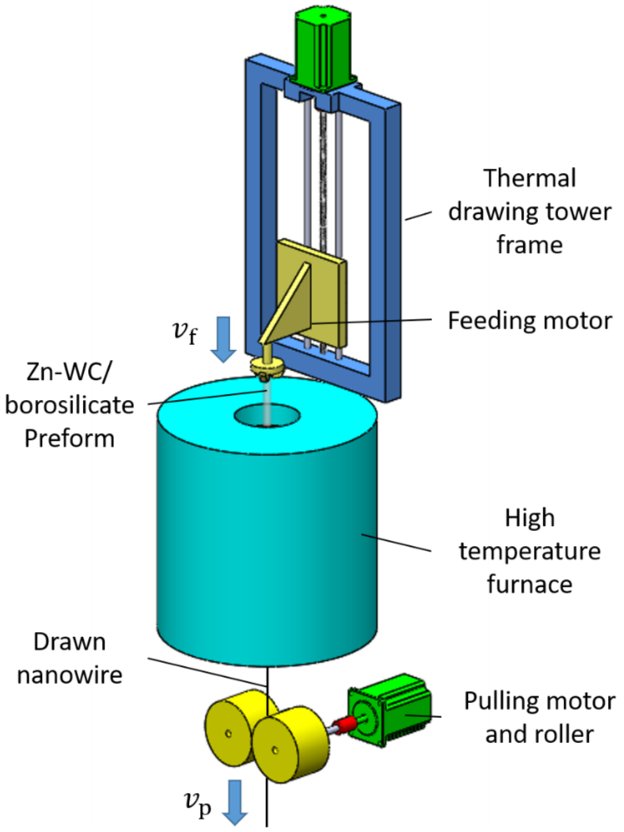

Supplementary Figure 4. Zn-WC microwires polished side view image acquired from SEM. WC nanoparticles (bright phases within the microwire) were dispersed homogeneously.

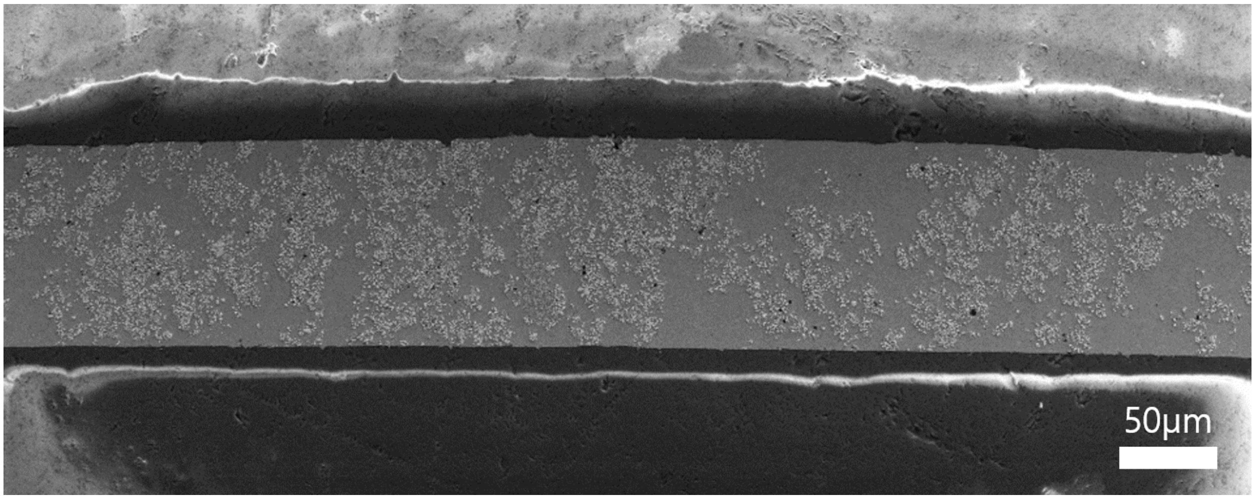

Supplementary Figure 5. Continuity characterization of Zn nanowire by optical microscopy. Zn has shown metallic reflectivity under microscopy, and droplet was not observed.

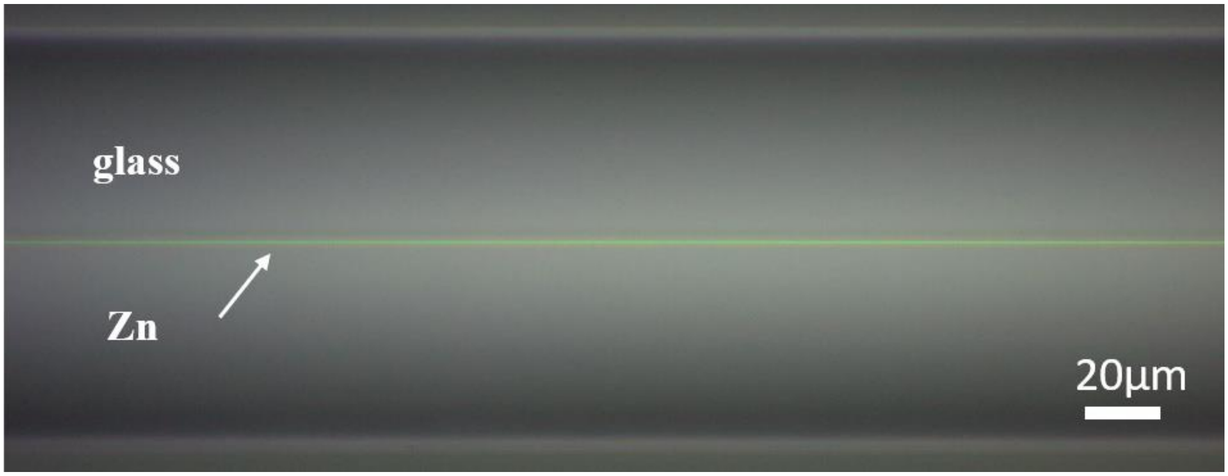

Supplementary Figure 6. Continuity characterization of Zn nanowire by electric conductivity. (a) The fiber segment was mounted in epoxy. (b) The sample surface was polished with wire tip exposed. Au/Pt deposition was performed on both surfaces to enable conductivity. (c) The resistive measurements between two faces of the epoxy on the sample with nanowire segments.

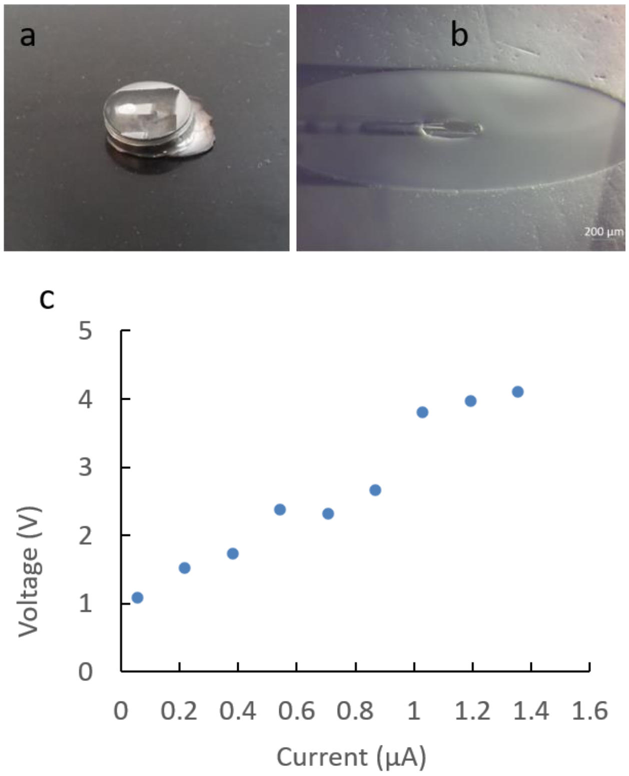

Supplementary Figure 7. Nanoparticle pinning effect for instability control. (a) The function plot of $(1-x^2)\Phi\left(x,\frac{\mu_{\text{core}}}{\mu_{\text{clad}}}\right)$ for $\frac{\mu_{\text{core}}}{\mu_{\text{clad}}}=10^6$, corresponding to the viscosity of Zn-10WC/borosilicate at 1100 K. (b) The demonstration varicose perturbation wave of long wavelength (~ 20 μm) corresponds to higher G value and lower τ', when there are no nanoparticles (c) The demonstration varicose perturbation wave of short wavelength (~ a few hundred nanometers) with nanoparticle pinning effect corresponds to lower G value and higher τ'.

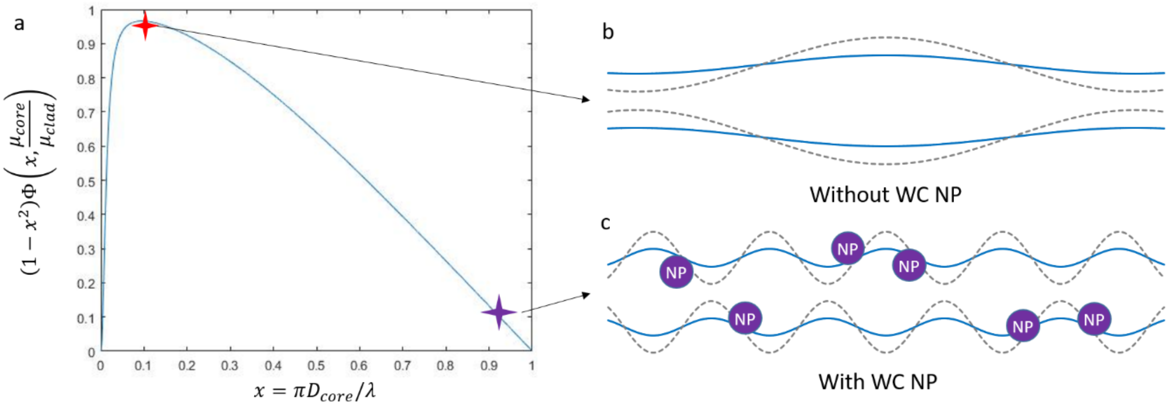

Supplementary Table 1. Viscosity measurement of molten Zn-WC. The viscosity of molten Zn and Zn-10WC were measured using a Modified Capillary Method based on the Hagen-Poisseulle formula of capillary flow[1].

	Capillary radius (mm)	Capillary length (cm)	Metal volume (mm^3)	Time (s)	Pressure difference (kPa)	Viscosity (mPa·s)
Zn	0.635	30	63.34	0.03	60	6
Zn-10WC	0.635	30	50.67	0.47	60	118

Supplementary Table 2. Representative micro/nanowire fabricated by thermal drawing. Interfacial energy and viscosity of the liquid materials are estimated by [2-6].

	Material (core/ cladding)	Fabrication temperature (°C)	Viscosity [Pa·s]	Interfacial energy [mJ/m^2]	Associated instability growth time [s/nm]	Ref.
Semiconductors	As_2Se_3/ PES	330	1.00E+04	93.3	2.44E+07	7
	Se/ PSU	240	4.67E-01	90.4	1.11E+07	8
	Se/ PES	240	4.67E-01	104.7	9.61E+06	9
Metal (Low T_m)	$SnAg_5$/ PES	240	1.88E-03	325.7	3.07E+06	9
	Sn/ Borosilicate	240	1.88E-03	439.7	2.28E+06	10
	Bi/ Borosilicate	820	1.03E-03	340.0	2.94E+06	11
	Ge/ Vycor	938	7.76E-04	460.5	2.17E+06	11
Metal (High T_m)	Au/ Fused Silica	1950	3.16E-03	1324.8	7.55E+05	12
	Cu/ Fused Silica	1880	1.99E-03	1501.6	6.66E+05	13
	Zn/ Borosilicate	850	3.35E-03	653.7	1.53E+06	10
	Zn-10WC/ Borosilicate	820	1.06E-02	130.6	7.68E+06	This work

Supplementary Table 3. Thermal drawing parameters and dimensions of preform/drawn wires.

Cycle	Preform core diameter (μm)	Preform cladding diameter (mm)	Feeding speed (mm/s)	Pulling speed (mm/s)	Drawdown ratio (D_r)	Expected core diameter of drawn wires (μm)	Drawing temperature (ºC)
1	1000	6.5	0.1	19.6	196	71	820
2	71	5.5	0.1	40	400	3.55	820
3	3.5	5.5	0.1	40	400	0.178	820

Supplementary Table 4. Typical metal nanowire (diameter less than 1 μm) manufacturing methods and results from the literature.

	Core material	Cladding	core diameter	length	reference
Thermal drawing	Au	Fused silica	260 nm	20 μm	[14]
Laser drawn-cast	Pd	Fused silica	250 nm	140 mm	[15]
	Pt	Quartz	10 nm	3 mm	[16]
	Au	Quartz	40 nm	2.7 mm	[16]
Electrospinning	Cu	N/A	50-200 nm	100 μm	[17]
	Au	N/A	400 nm	10 mm	[18]
	Ni	N/A	120 nm	>20 μm	[19]
Chemical synthesis	Ag	N/A	60 nm	100 μm	[20]
	Cu	N/A	90-120 nm	40-50 μm	[21]
	Au	N/A	16-66 nm	10 μm	[22]
Nanoparticle controlled thermal drawing	Zn-WC	Pyrex	170 nm	31 mm	This work

Supplementary Note 1: Tomotika model of fluid instability and thermal drawing.

Tomotika dispersion relation considers a long cylindrical thread of a viscous liquid (core material) in an infinite mass of another viscous fluid (cladding material), under the supposition that there are no general flows in both fluids, giving small disturbances[23]. This model assumes a sysmetrical perturbation motion about the axial direction is proportional to an exponentially amplifying sinusoidal wave. The term associated instability growth time (τ') can be generally understood as a measurement of time-lapse in which the core would remain continuous before it is broken into droplets (referring to the wave amplitude larger than the core thread radius). According to the Tomotika model, which describes the fluid instability in cladding under ideal static conditions (e.g., small motion, no interface slipping, negligible fluid flow, and interfacial surface tension as the only normal stress), the instability growth time is related to the core diameter (D), cladding viscosity (μ_{clad}), interfacial energy (γ), and G. G is the maximum of a function $(1 - x^2)\Phi(x)$, implying the maximum instability at a predefined viscosity ratio ($\mu_{\text{core}}/\mu_{\text{clad}}$), shown in Supplementary Fig. 5. Under the ideal static condition, the maximum instability can induce a varicosity wavelength, according to equation (2), implying the case where the liquid thread is easiest to break. Without additional boundary conditions, the varicosity wavelength is generated corresponding to G and x.

In experiments, the cladding viscosity is a parameter that could be tuned by temperature, which has its upper limit at the glass transition temperature. Moreover, when drawing at the glass transition temperature, the high viscosity of glass cladding would inhibit the flowability and significantly reduces the productivity and the scalability, which are supposed to be one of the most significant advantages of thermal drawing. Furthermore, additional influential factors of drawing speed and longitudinal stretching force will need to be considered in the actual drawing process for optimization[24].

The experimental time of thermal drawing is described as

$$t = \frac{L}{v_{\text{p}}} \tag{1}$$

where v_{p} is the pulling speed of the thermal drawing, L is the effective length of the furnace where metal core remains liquid, starting from the bottom of the taper to the bottom of the furnace. L is 12.5cm in the setup in this study, approximately half of the furnace length.

Supplementary Note 2: Nanoparticle-enabled control of viscosity and interfacial energy.

Supplementary Equation (2) refers to the empirical correlation of the nanocomposite viscosity[25]. Supplementary Equation (3) estimates decreased nanocomposite surface tension, based on the Ni-Al_2O_3 system[26]. Supplementary Equation (4) refers to Young's equation to estimate the interfacial energy of Zn-WC/borosilicate. The interfacial energy of Zn-10WC/ borosilicate was calculated using the measured contact angle from Supplementary Fig. 2, and the result was indicated in Supplementary Table 1. The interfacial energy reduction could be the improvement of the wettability since nanoparticles in the metal reduced the metallic bonds in the base metal such that the surface tension decreased. Although the viscosity and interfacial energy of the Zn-WC system has never been studied before, correlations from similar nanocomposite system could still provide an insightful estimation on nanoparticles effects to the Zn melt properties.

$$\frac{\mu_{\text{eff}}}{\mu_0} = \frac{1}{1 - 34.87\left(d_p/d_f\right)^{-0.3}\varphi^{1.03}} \quad (2)$$

$$\frac{\gamma_{\text{eff}}}{\gamma_0} = -3.7344\varphi + 1 \quad (3)$$

$$\gamma_{\text{sl}} = \gamma_{\text{eff}} - \gamma_{\text{l}} \cos\theta \quad (4)$$

where, μ_{eff}, μ_0 and γ_{eff}, γ_0 are respectively the viscosities and surface tensions of molten nanocomposites and pure molten metals, d_p is the nanoparticle size, d_f is the metal atom size, φ is the nanoparticle volume concentration, γ_{sl} is the interfacial energy between Zn-WC (liquid) and borosilicate (solid), γ_{l} is the surface tension of the borosilicate, and θ is the contact angle.

Supplementary Note 3: Nanoparticle Pinning Effects to Overcome Fluid Instability at Nanoscale.

First, nanoparticles at the Zn-borosilicate interface could induce lower interfacial energy locally for a stabilization effect. SEM and TEM images showed that WC nanoparticles move from inside the metal wire to the Zn-borosilicate interface during the thermal drawing process. The results indicated that WC nanoparticles would be more stable at the Zn/borosilicate interface due to the minimization of interfacial energy[27] and favorable wettability between molten Zn and WC[28]. Furthermore, nanoparticles would not move much since the glass cladding is in a highly viscous state. The new interface of WC/Zn, obtaining low interfacial energy at the points of contacts, could efficiently increase τ' for fluid instability suppression. Such a phenomenon of nanoparticle staying at the viscous liquid surface for modifying the interfacial properties of the ionic liquid was also observed in related applications[29].

Second, nanoparticles at the liquid thread surface could act as boundary pinning points to the varicose perturbation waves, namely a pinning effect. In the experimental condition, where the viscosity ratio is approximately 10^6, the varicosity wavelength is 21 µm, indicated by the red star shown in Supplementary Fig. 5a. Consequently, the nanowires break at this wavelength corresponding to the maximum instability, directly related to G, as demonstrated in Supplementary Fig. 5b. When nanoparticles served as boundary pinning points on the surface of liquid Zn, the non-ideal condition constrained the varicosity wavelength to a smaller value, shown in Supplementary Fig. 5c. The wavelength is approximately the same magnitude of the local distance between adjacent nanoparticles, typically a few hundreds of nanometers (Fig. 3c and 3e). A much short wavelength λ resulted in a relatively low G value (indicated by the purple star) and the corresponding high τ' (in addition to the contribution from low interfacial energy), shown in Fig. 5c. Although such wavelength reduction was technically uneasy to observe, the observation of nanoparticles on nanowire surfaces supported the hypothesis that nanoparticles induced pinning effects to suppress the fluid instability by acting as boundary pinning points to reduce the varicosity wavelength to increase τ'.

Supplementary References

1 Gancarz, T., Moser, Z., Gąsior, W., Pstruś, J. & Henein, H. A comparison of surface tension, viscosity, and density of Sn and Sn–Ag alloys using different measurement techniques. *Int. J. Thermophys.* **32**, 1210-1233 (2011).

2 Lu, H. & Jiang, Q. Surface tension and its temperature coefficient for liquid metals. *J. Phys. Chem. B* **109**, 15463-15468 (2005).

3 Nogi, K., Ogino, K., McLean, A. & Miller, W. The temperature coefficient of the surface tension of pure liquid metals. *Metall. Tran. B* **17**, 163-170 (1986).

4 Tegetmeier, A., Cröll, A. & Benz, K. A formula describing the temperature dependence of surface tension for some semiconductors melts. *J. Cryst. Growth* **141**, 451-454 (1994).

5 Bircumshaw, L. CXXIV. The surface tension of liquid metals.—Part II. The surface tension of bismuth, cadmium, zinc, and, antimony. *Lond. Edinb. Dubl. Phil. Mag.* **3**, 1286-1294 (1927).

6 Koštál, P. & Málek, J. Viscosity of selenium melt. *J Non-Cryst. Solids* **356**, 2803-2806 (2010).

7 Kaufman, J. J., Tao, G., Shabahang, S., Deng, D. S., Fink, Y. & Abouraddy, A. F. Thermal drawing of high-density macroscopic arrays of well-ordered sub-5-nm-diameter nanowires. *Nano Lett.* **11**, 4768-4773 (2011).

8 Deng, D., Orf, N., Danto, S., Abouraddy, A., Joannopoulos, J. & Fink, Y. Processing and properties of centimeter-long, in-fiber, crystalline-selenium filaments. *Appl. Phys. Lett.* **96**, 023102 (2010).

9 Yaman, M., Khudiyev, T., Ozgur, E., Kanik, M., Aktas, O., Ozgur, E. O., Deniz, H., Korkut, E. & Bayindir, M. Arrays of indefinitely long uniform nanowires and nanotubes. *Nat. Mater.* **10**, 494-501 (2011).

10 Zhang, X., Ma, Z., Yuan, Z. Y. & Su, M. Mass-productions of vertically aligned extremely long metallic micro/nanowires using fiber drawing nanomanufacturing. *Adv. Mater.* **20**, 1310-1314 (2008).

11 Badinter, E., Ioisher, A., Monaico, E., Postolache, V. & Tiginyanu, I. Exceptional integration of metal or semimetal nanowires in human-hair-like glass fiber. *Mater. Lett.* **64**, 1902-1904 (2010).

12 Ioisher, A., Badinter, E., Monaico, E., Postolache, V., Hartnagel, H. L., Leporda, N. & Tiginyanu, I. Integration of Ge nanowire arrays in glass micro-fibers. *Surf. Engin. Appl. Electrochem.* **47**, 4 (2011).

13 Hou, J., Bird, D., George, A., Maier, S., Kuhlmey, B. T. & Knight, J. Metallic mode confinement in microstructured fibres. *Opt. Express* **16**, 5983-5990 (2008).

14 Tyagi, H., Lee, H., Uebel, P., Schmidt, M., Joly, N., Scharrer, M. & Russell, P. S. J. Plasmon resonances on gold nanowires directly drawn in a step-index fiber. *Opt. Lett.* **35**, 2573-2575 (2010).

15 Zhang, K., Han, K., Shi, S., Bahl, G. & Tawfick, S. Highly Stretchable Conductors Made by Laser Draw-Casting of Ultralong Metal Nanowires. *Adv. Electron. Mater.* **2**, 1600003 (2016).

16 Percival, S. J., Vartanian, N. E. & Zhang, B. Laser-pulled ultralong platinum and gold nanowires. *RSC Adv.* **4**, 10491-10498 (2014).

17 Wu, H., Hu, L., Rowell, M. W., Kong, D., Cha, J. J., McDonough, J. R., Zhu, J., Yang, Y., McGehee, M. D. & Cui, Y. Electrospun metal nanofiber webs as high-performance transparent electrode. *Nano Lett.* **10**, 4242-4248 (2010).

18 Wu, H., Kong, D., Ruan, Z., Hsu, P.-C., Wang, S., Yu, Z., Carney, T. J., Hu, L., Fan, S. & Cui, Y. A transparent electrode based on a metal nanotrough network. *Nature nanotechnology* **8**, 421-425 (2013).

19 Barakat, N. A., Kim, B. & Kim, H. Y. Production of smooth and pure nickel metal nanofibers by the electrospinning technique: nanofibers possess splendid magnetic properties. *J. Phys. Chem. C* **113**, 531-536 (2009).

20 Jiu, J., Araki, T., Wang, J., Nogi, M., Sugahara, T., Nagao, S., Koga, H., Suganuma, K., Nakazawa, E. & Hara, M. Facile synthesis of very-long silver nanowires for transparent electrodes. *J. Mater. Chem. A* **2**, 6326-6330 (2014).

21 Chang, Y., Lye, M. L. & Zeng, H. C. Large-scale synthesis of high-quality ultralong copper nanowires. *Langmuir* **21**, 3746-3748 (2005).

22 Kim, F., Sohn, K., Wu, J. & Huang, J. Chemical synthesis of gold nanowires in acidic solutions. *J. Am. Chem. Soc.* **130**, 14442-14443 (2008).

23 Tomotika, S. On the instability of a cylindrical thread of a viscous liquid surrounded by another viscous fluid. *Proc. R. Soc. Lond. A* **150**, 322-337 (1935).

24 Eggers, J. & Villermaux, E. Physics of liquid jets. *Rep. Prog. Phys.* **71**, 036601 (2008).

25 Corcione, M. Empirical correlating equations for predicting the effective thermal conductivity and dynamic viscosity of nanofluids. *Energy Convers. Manag.* **52**, 789-793 (2011).

26 Ma, C., Zhao, J., Cao, C., Lin, T.-C., Li, X. & Engineering. Fundamental Study on laser interactions with nanoparticles-reinforced metals—part II: effect of nanoparticles on surface tension, viscosity, and laser melting. *J. Manuf. Sci. Eng.* **138** (2016).

27 Xu, J., Chen, L., Choi, H., Konish, H. & Li, X. Assembly of metals and nanoparticles into novel nanocomposite superstructures. *Sci. Rep.* **3**, 1730 (2013).

28 Hwang, I., Guan, Z. & Li, X. Fabrication of zinc–tungsten carbide nanocomposite using cold compaction followed by melting. *J. Manuf. Sci. Eng.* **140** (2018).

29 Ravera, F., Santini, E., Loglio, G., Ferrari, M. & Liggieri, L. Effect of nanoparticles on the interfacial properties of liquid/liquid and liquid/air surface layers. *J. Phys. Chem. B* **110**, 19543-19551 (2006).
